# Regeneration and Union of Nerves

**Published:** 1842-05

**Authors:** 


*Regeneration and Union of Nerves.*—By MM. Gunther and Schon. The following observations were made on rabbits, about fifty of which were operated on, sometimes by cutting across the ischiatic nerves, at other times by removing a portion varying from two to four lines in length. These physiologists describe the elementary fibres of the nerves as transparent cylinders with double tunics, filled with a fluid resembling liquid albumen. After maceration in water, or after death, this fluids coagulates and produces the turbid granular aspect which till now, has been considered its natuural appearance. When the two extremities of a nerve which has been cut across become united, the nerve propagates impression through the uniting medium. This is done by means of true primary fibres having been formed through the uniting medium, and the following is the mode in which MM. Gunther and Schon have observed this to take place. After division of the nerve the two ends retract somewhat, the diameter of the neurilema becomes diminished, and the medullary substance is pushed out in a globular form; exudation of plastic lymph then occurs and fills the wound, and the cut extremities of the nerve become swollen, the upper extremity more than the latter. This tumefaction of the nerve itself, is owing to the presence of plastic lymph effused into the cellular tissue and also between the neurilema and primitive fibres.

The matter which unites the two ends of the nerve is at first amorphous, but by degrees primitive fibres shoot through the mass, and become visible at the earliest on the eighth week after the division. These new fibres are in every respect similar to the original fibres; but the granular exudation and the cellular tissue which surround and envelope them render them difficult of examination. These fibres are not parallel, but exhibit a confused arrangement.

With the regeneration of the fibres of the nerve returns the sensibility and mobility of the limb or organs to which the nerve was distributed.

But in general the function of the part is not so free as before the division:—the animals not being able to use the limb whose nerve had been divided so freely as the other. The influence of the will over the limb was also observed to be diminished. MM. Gunther and Schon account for this on the supposition which is borne out by their experiments, that the number of fibres which are regenerated is not equal to those which originally existed; besides, it appeared to them that the regenerated fibres occasionally united the ends of different primitive fibres, so that the sensation was not always referred to its proper place. They consider that the regeneration or growth of the uniting fibre commenced at the superior extremity of the divided nerve, but concede that it is possible that it may also take place from the lower extremity.—*Ed. Med. & Surg. Journ.* Jan. 1842, from *Archives Generales*, March, 1841.

